# Mitochondria‐associated endoplasmic reticulum membrane: Overview and inextricable link with cancer

**DOI:** 10.1111/jcmm.17696

**Published:** 2023-02-27

**Authors:** Xi Yang, Jing Zhuang, Weilong Song, Wangjie Shen, Wei Wu, Hong Shen, Shuwen Han

**Affiliations:** ^1^ Huzhou Central Hospital, Affiliated Central Hospital Huzhou University Huzhou China; ^2^ Key Laboratory of Multiomics Research and Clinical Transformation of Digestive Cancer of Huzhou Huzhou China; ^3^ School of Medicine Huzhou University Huzhou China

**Keywords:** autophagy, calcium transfer, cancer, endoplasmic reticulum, ER stress, lipid synthesis, mitochondria, mitochondrial‐associated membrane

## Abstract

The mitochondrial‐associated membrane (MAM) is a physical platform that facilitates communication between the endoplasmic reticulum (ER) and mitochondria. It is enriched with many proteins and enzymes and plays an important role in the regulation of several fundamental physiological processes, such as calcium (Ca^2+^) transfer, lipid synthesis, cellular autophagy and ER stress. Accumulating evidence suggests that oncogenes and suppressor genes are present at the ER‐mitochondrial contact site, and their alterations can affect Ca^2+^ flux, lipid homeostasis, and the dysregulation of mitochondrial dynamics, thereby influencing the fate of cancer cells. Understanding the fundamental role of MAM‐resident proteins in tumorigenesis could support the search for novel therapeutic targets in cancer. In this review, we summarize the basic structure of MAM and the core functions of MAM‐resident proteins in tumorigenesis. In addition, we discuss the mechanisms by which natural compounds promote cancer cell apoptosis from the perspective of ER stress.

## 1 INTRODUCTION

Cancer is currently considered one of the biggest public health problems. Owing to its high incidence, prevalence and mortality, it has a huge economic impact on every health system worldwide. It is estimated that by 2040, the number of cancer cases will have reached 28 million, with 16 million patients dying from this disease.^1^ Its pathology is driven by uncontrolled cell proliferation and an increased dysregulation of important cellular pathways.^2^ Thus, reprogramming metabolic requirements and energy metabolism is a hallmark of cancer, and the key signalling pathways involved in these processes have emerged as important therapeutic targets.^3^

The metabolism of cancer cells often involves aerobic glycolysis, a phenomenon known as the ‘Warburg effect’.^4^ Currently, most cancer cells rely on mitochondrial metabolism and use large amounts of glucose‐derived pyruvate to generate ATP through oxidative phosphorylation (OXPHOS), suggesting that mitochondria are key players in cancer biology. There is increasing evidence that calcium (Ca^2+^) is critical for mitochondrial metabolism; in particular, Ca^2+^ communication between the ER and mitochondria is key to mitochondrial function, as well as cancer progression.^5^ Moreover, the dysregulation of lipid metabolism is a significant metabolic alteration in cancer. Cancer cells in the tumour microenvironment can utilize lipid metabolism to support rapid proliferation, migration, invasion and survival.^6^ As cancer cells continue to divide, spread and form solid tumours, increased hypoxia and nutrient deprivation in the environment may promote cancer cell death and prevent their growth and progression. Autophagy is critical for cell survival and the maintenance of tumorigenesis under these stress conditions.^7^ Notably, autophagy may be affected by disturbed Ca^2+^ homeostasis and starvation; that is, signals from Ca^2+^ perturbation and glucose transport blockade may be involved in ER stress activation, which is one of the possible pathways to induce autophagy.^8^ A previous study revealed that ER stress can effectively induce autophagy in tumour cells in various malignant tumours.^9^ Taken together, understanding the basic physiological processes involved in Ca^2+^ homeostasis, lipid metabolism, autophagy and ER stress can provide a theoretical basis for tumorigenesis.

The ER and mitochondria play crucial roles in regulating cellular homeostasis and cell death. Researchers have found that they constantly exchange information at contact sites called the mitochondria‐associated ER membrane (MAM).^10^ Currently, it is generally accepted that MAM is a 10–25 nm wide region (approximately 2%–5% of the mitochondrial surface) juxtaposed by the ER and mitochondrial membranes tethered together by proteins, without the complete fusion or loss of organelle properties.^11^ With further studies on the cell‐specific MAM proteome and the application of new technologies, increasing evidence has revealed that MAM is a hub for signalling molecules and pathways that control and regulate cellular homeostasis.^12^ Szymański et al.^13^ demonstrated that MAM is involved in a variety of fundamental biological processes, such as regulation of Ca^2+^ homeostasis, lipid metabolism, autophagy and ER stress. Thus, MAM is critical for maintaining cellular health, and its disruption can lead to the dysfunction of various cellular processes.

**FIGURE 1:**
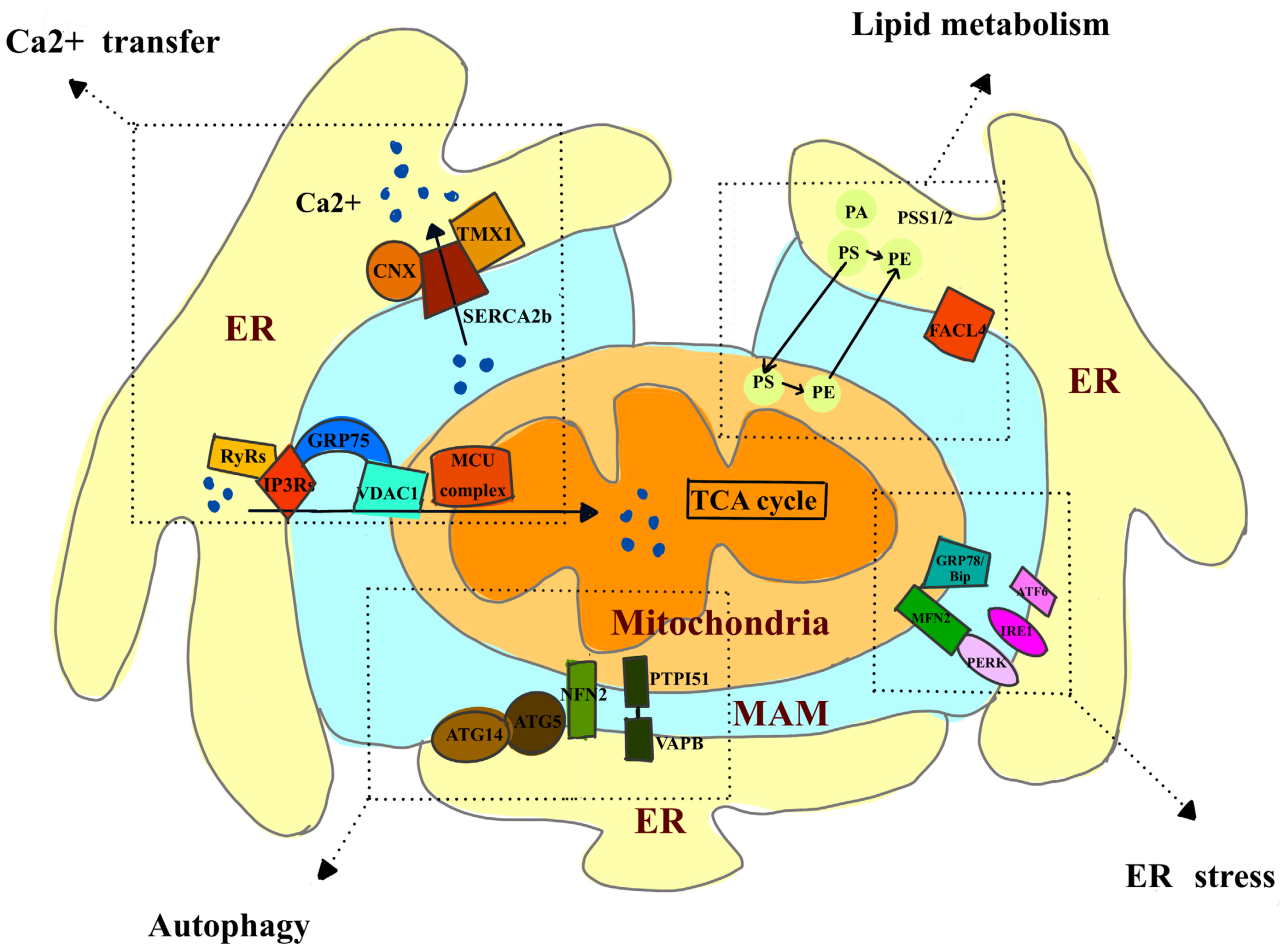
Schematic summary of key MAM‐resident proteins involved in ER‐mitochondria Ca^2+^ transfer, lipid metabolism, autophagy and ER stress. ER, endoplasmic reticulum; MAM, mitochondrial‐associated membrane.

In the last decade, advances in the characterization of the mechanisms underlying tumorigenesis have shifted the focus to the structure of MAM. Recent studies have clearly demonstrated that abnormalities in MAM are important mechanisms involved in cancer pathology.^14^ In this review, we discuss emerging evidence that elucidates the important biological processes that regulate ER‐mitochondrial juxtaposition, and how cancer cells can use these processes to support their malignant phenotype. The key MAM‐resident proteins involved in these biological processes are summarized in Figure 1.

## 2 STRUCTURE AND COMPOSITION OF MAM

**FIGURE 2:**
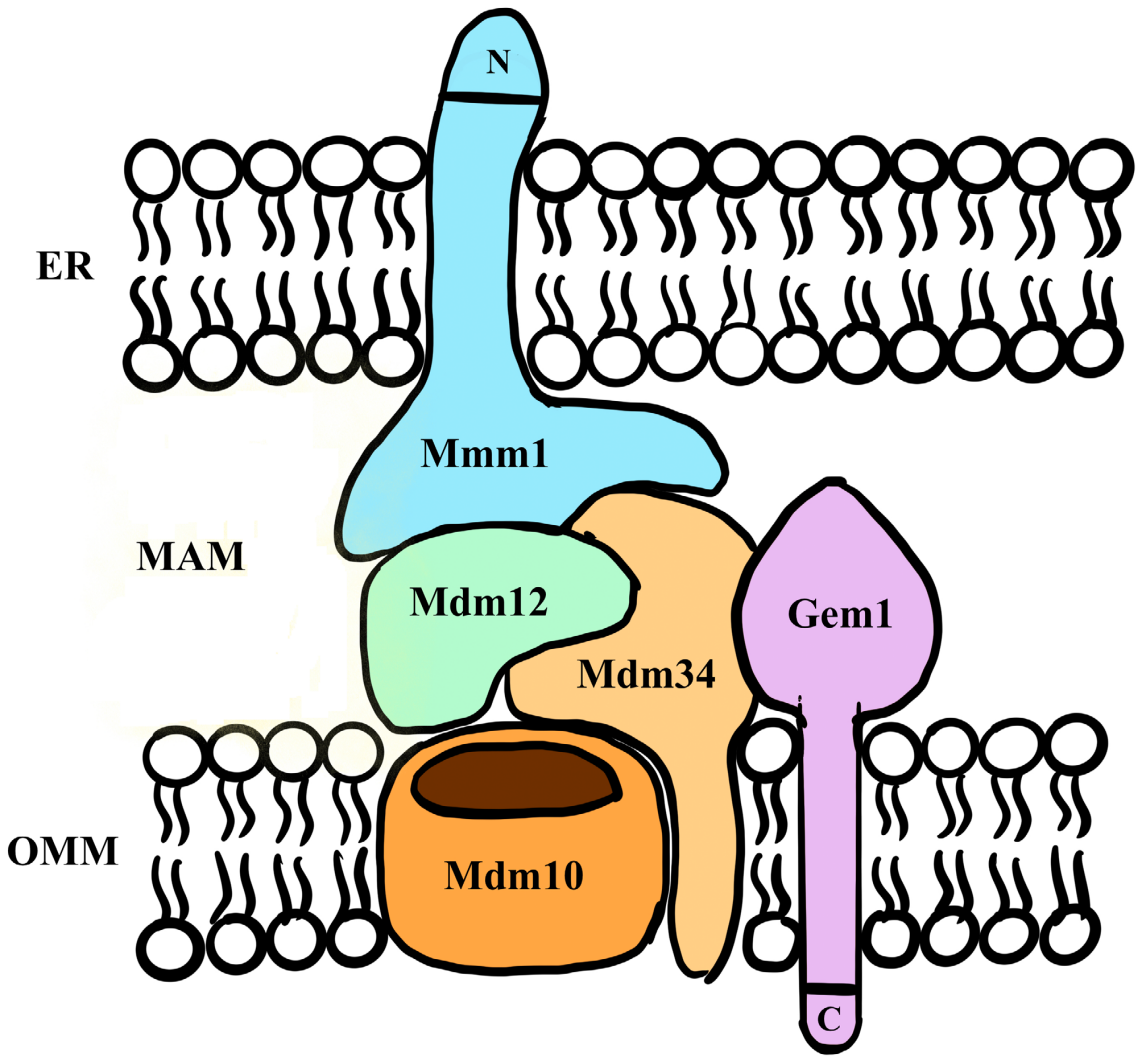
Schematic model of the ERMES complex. ERMES is composed of five subunits, including the mitochondrial outer membrane protein Mdm10, ER‐resident protein Mmm1, and three peripheral membrane proteins, namely Mdm34, Mdm1 and Gem1. ER, endoplasmic reticulum; ERMES, endoplasmic reticulum and mitochondria encounter structures.

In recent years, techniques for the biochemical isolation of MAM from different cells and tissues have been optimized, leading to great advances in the field of MAM. At the same time, the identification of endoplasmic reticulum and mitochondria encounter structures (ERMES) has led to a deeper understanding of their biochemical composition and functions. In *Saccharomyces cerevisiae*, a complex of Mdm10, Mmm12, Mdm34, and Mdm1 has been identified as a molecular chain between the ER and mitochondria.^15^ The components of this complex may be localized to the ER and mitochondria, which may serve to link organelles together. Furthermore, Stroud et al.^16^ identified the GTPase Gem1 as a novel ERMES subunit. Together, ERMES is composed of five subunits: the mitochondrial outer membrane protein Mdm10, ER‐resident protein Mmm1 and three peripheral membrane proteins, namely Mdm34, Mdm1 and Gem1 (Figure 2). Mmm1 consists of an N‐terminal and a C‐terminal domain and is separated by transmembrane fragments formed by amino acids 92–116.^17^ Mass spectrometry analysis has determined that Mmm1 is glycosylated only in the N‐terminal region and is localized to the official lumen of the ER membrane.^18^ Its C‐terminal cytoplasmic structural domain interacts with other ERMES subunits associated with the mitochondria and is essential for ERMES function.^16^ The β‐barrel protein Mdm10 resides mainly in the outer mitochondrial membrane (OMM),^19^ and the interaction between Mmm1 and Mdm10 requires the involvement of Mdm12 and Mdm34 for regulation.^20^ In addition, Gem1 carries a transmembrane fragment at the C‐terminus and potentially correlates with the regulation of intracellular calcium ion concentrations by ERMES.^21^ Thus, MAM forms ER‐mitochondrial junctions that integrate components involved in central organelle function and may provide a platform for coordinating mitochondrial biogenesis and membrane dynamics.

## 3 Ca^2+^ TRANSFER AND MAM

### 3.1 Ca^2+^ transfer at the ER‐mitochondria interface

Ca^2+^ signals are usually derived from the ER and lysosomes^22^ and affect other organelles through microdomains established at membrane contact sites.^23^ It has been shown that an important target organelle for ER‐derived Ca^2+^ signalling is the mitochondrion, which contributes to the maintenance of intracellular Ca^2+^ homeostasis.^24^ In fact, Ca^2+^ is released from the ER, passes across the two mitochondrial membranes OMM and inner mitochondrial membrane (IMM) and is then pumped back to the ER. In this process, ER‐mitochondrial binding participates in Ca^2+^ transport with the help of numerous MAM‐ and OMM‐resident proteins.

Two important ER‐resident proteins are involved in Ca^2+^ transport and are enriched in ERMES: sarco/ER Ca^2+^ ATPase (SERCA) and inositol 1,4,5‐trisphosphate receptor (IP3R). The SERCA pump localizes to the ER membrane and is responsible for pumping Ca^2+^ from the cytoplasm into the ER, thus forming a Ca^2+^ gradient between the cytoplasm and ER to regulate ER Ca^2+^ levels.^25^ In general, the SERCA pump is encoded by three genes (SERCA1, 2 and 3),^26^ among which the SERCA2b (a variant of SERCA2) isoform showed the highest Ca^2+^ affinity.^27^ Two important proteins enriched in MAM, calnexin and thioredoxin‐related transmembrane protein 1 (TMX1), have been reported to interact directly with SERCA2b.^28^, ^29^ In brief, palmitoylation of calnexin induces a functional transition from the quality control of protein folding to ER‐Ca^2+^ signalling by enhancing SERCA2b activity, while TMX1 counteracts this interaction and suppresses SERCA2b activity, thereby promoting the inward flow of Ca^2+^ to the mitochondria.^29^

Mechanistically, ryanodine receptors (RyRs) (primarily expressed in the sarcoplasmic reticulum) and IP3Rs form a tetrameric channel that controls the release of ER Ca^2+^.^30^, ^31^ In vertebrates, there are three IP3R isoforms (IP3R1–3), of which IP3R3 (enriched in the MAM) serves as a major regulator of Ca^2+^ signalling to the mitochondria.^32^ In addition, IP3R3 interacts with mitochondrial voltage‐dependent anion channel 1 (VDAC1) residing in the OMM and is connected via the molecular chaperone glucose regulator 75 (GRP75), thereby promoting Ca^2+^ uptake by the OMM.^24^ Furthermore, since the IMM is not ion permeable, the mitochondrial calcium uniporter (MCU) transports Ca^2+^ to the mitochondrial matrix.^33^ MCU is a multiprotein complex composed of MCU1, MCU2, MCUb, EMER, MCUR1 and SLC25A3, among others.^34^ MICU1 is considered the most representative Ca^2+^ uptake regulatory protein and functions as a gatekeeper for sensing Ca^2+^ levels in the intermembrane space.^35^ In general, at low concentrations, the gate is closed. However, once the Ca^2+^ levels exceed the threshold (≥500 nM),^36^ the Ca^2+^ binding site of MICU1 undergoes a conformational change, thus opening the channel. Together, the MCU multiprotein complex tightly controls Ca^2+^ concentration in the mitochondrial matrix, thereby regulating cell metabolism, survival and death.

### 3.2 Roles of ER‐mitochondria Ca^2+^ signalling in tumorigenesis

In tumours, alterations in the cellular microenvironment are usually associated with the activation of transmembrane receptors and Ca^2+^ permeabilization channels, thereby altering Ca^2+^ homeostasis. In this section, we discuss the dysregulation of Ca^2+^ homeostasis in cancer, centred on MAM.

The abnormal expression of MAM‐resident proteins in cancer has been widely reported, for example, sigma‐1 receptor is highly expressed in cancer cells compared with normal tissues.^37^, ^38^ As mentioned above, IP3R is a central target for controlling Ca^2+^ transfer between the ER and mitochondria and is actively involved in the key processes of tumorigenesis in various tumours. There are three known isoforms of IP3R (IP3R1–3), among which IP3R1 and IP3R2 have been found to be overexpressed in both non‐cancer and colon cancer tissues. On the contrary, IP3R1 was only been detected in intracellular pathways in cancer cells, and its expression is correlated with cell invasion and metastasis. The silencing of intracellular IP3R3 was found to result in increased levels of apoptosis in ovarian cancer A2780 and CRC DLD1 cell lines.^39^ In gastric cancer, IP3R3 was weakly expressed in the primary tumour cell line and overexpressed in cancer cell lines established by malignant ascites.^40^ In addition, IP3R3 was overexpressed in hepatocellular carcinoma (HCC) tissues from patient cohorts and demethylated orthotopic HCC mouse models. Overexpressed IP3R3 expression led to enhance intracellular Ca^2+^ signalling, reduce apoptosis and shorten overall survival time of patients with HCC.^41^ Interestingly, several oncogenes and tumour suppressors are located in MAM and interact with IP3R to alter Ca^2+^ release patterns and cancer cell fate. For example, the proto‐oncogene serine/threonine kinase (Akt) mediates the phosphorylation of IP3R3, thereby inhibiting ER Ca^2+^ flux and apoptosis. Alterations in the Akt pathway have been reported to promote tumorigenesis and are overexpressed in various tumours, such as colorectal cancer (CRC), HCC and pancreatic cancer.^42^ Moreover, mammalian target of rapamycin 2 (mTORc2, an oncogene) is localized to MAM in a growth factor‐stimulated manner. A previous study found that mTORc2 phosphorylates and activates Akt, which mediates Ca^2+^ flux, energy metabolism and MAM integrity.^43^, ^44^ Another study reported that mTORc2 promotes tumorigenesis by regulating the Akt protein in HCC.^45^ In addition, the dysregulation of Bcl‐2 is a key factor in distinguishing normal cells from cancer cells.^46^ Bcl‐2 family members (Bcl‐2 and Bcl‐xl) in the ER regulate IP3R activity and apoptosis by controlling cytochrome C release and the activation of cysteine aspartate proteases. In ovarian cancer, Bcl‐2 knockdown promotes high ER Ca^2+^ transport to mitochondria through MAM, leading to mitochondrial Ca^2+^ overload, which increases calpain‐1‐mediated apoptosis.^47^ In addition, the overexpression of the Bcl‐2 gene has been detected in non‐small cell lung cancer (NSCLC), and Bcl‐2 protected cancer cells from apoptosis by isolating members of the pro‐apoptotic family and regulating IP3R‐mediated Ca^2+^ signalling.^48^ In contrast to Bcl‐2, Bcl‐xl has a sensitizing effect on IP3Rs.^49^ Interestingly, Bcl‐xl also suppresses IP3R‐mediated Ca^2+^ release via interactions with the same IP3R region as Bcl‐2. In a variety of cell systems, such as breast cancer cells, Bcl‐xl inhibits Ca^2+^ release from the ER to the cytoplasm, thereby protecting cells from IP3R‐mediated apoptosis.^50^ Moreover, Bcl‐xl can inhibit voltage‐dependent anion channel 1 (VDAC1)‐mediated mitochondrial Ca^2+^ uptake in breast cancer cells, which can alter mitochondrial ATP production and enhance reactive oxygen species (ROS) production, thus facilitating cancer cell migration.^51^

The sigma‐1 receptor (Sig‐1R), a chaperone protein enriched in MAM, plays a fundamental role in regulating Ca^2+^ transport mechanisms and leads to enhance cellular survival in response to environmental stress, especially in cancer cells.^52^ Sig‐1R is expressed at low levels in normal cells and is highly expressed in prostate, breast and lung cancers. Specifically, upregulated Sig‐1R expression was associated with a higher CRC grade and a lower prognosis in patients with breast cancer.^53^ Sig‐1R binds directly to the Ca^2+^‐activated potassium channel SK3 and drives its functional coupling to voltage‐independent Orai1 channels in the lipid nanostructure domain of the plasma membrane. Moreover, silencing Sig‐1R inhibited SK3: Orai1‐dependent Ca^2+^ inward flow in CRC and breast cancer cells, resulting in the inhibition of cell migration.^38^ Together, Sig‐1R‐related chaperone proteins control the electrical plasticity of cancer cells via ‘driving’ ion channels to enhance cancer cell proliferation, anti‐apoptosis and angiogenesis.^54^

Sarco/ER Ca^2+^ ATPase are the only channels in the ER responsible for Ca^2+^ uptake, and their functional activity is affected by oncogenes and tumour suppressors localized in the MAM, further regulating cancer cell fate by remodelling ER‐Ca^2+^ homeostasis.^55^ p53 is a common tumour suppressor gene that is mutated in approximately 50% of aggressive tumours. A previous study showed that the low expression of p53 in human colon cancer HCT‐116 cells is associated with a significant reduction in ER Ca^2+^ uptake and a reduced ability of Ca^2+^ to flow to the mitochondria.^56^ After the treatment of cancer cells with adriamycin (a chemotherapeutic drug), p53 activates and accumulates in the MAM region and rapidly binds to the SERCA pump to reduce its oxidation and increase SERCA activity, resulting in Ca^2+^ overload in the mitochondria to release caspase cofactors to induce apoptosis. Another candidate gene, TMX1, is also enriched in MAM and can negatively regulate SERCA2b function.^29^ An experimental study revealed that melanoma cells (A375P) with low expression of TMX1 showed increased ER Ca^2+^ and reduced Ca^2+^ translocation to mitochondria, indicating that low levels of TMX1 divert bioenergy away from mitochondria by reducing ER‐mitochondrial contact and accelerating tumour growth.^57^ In conclusion, the dysregulation of Ca^2+^ homeostasis in the tumour microenvironment is directly related to Ca^2+^ transport from ER to mitochondria while Ca^2+^ transport is regulated by Ca^2+^ transporter proteins, tumour suppressors and oncogenes. Dysregulation of Ca^2+^ homeostasis induced by different factors can promote cell apoptosis or survival to alter tumorigenesis. In addition, MAM is also important in tumorigenesis since it provides a platform for the dysregulation of Ca^2+^ homeostasis.

## 4 LIPID METABOLISM AND MAM

### 4.1 Lipid transport and synthesis at the ER‐mitochondria interface

Mitochondrial‐associated membrane is essential for many lipid metabolic pathways and is required for the communication of information between ER and mitochondria.^58^ Phosphatidylserine (PS) is synthesized in the ER by phosphatidylserine synthase (Pss1/2). The synthesized PS is transported into the mitochondria, where it is decarboxylated to form phosphatidylethanolamine (PE) by PS decarboxylase (PSD) in the IMM. PE is then rapidly exported to the ER, where it is further modified to PC by phosphatidylethanolamine *N*‐methyltransferase (PEMT2).^59^, ^60^ Previous evidence suggests that the inhibition of PSD activity causes a significant accumulation of PS in MAM.^61^ Indeed, PS transfer to mitochondria via MAM is the rate‐limiting step in PE generation and is, therefore, essential for maintaining phospholipid homeostasis between cells and mitochondria.^62^ In addition, mitochondria synthesize a signature lipid, cardiolipin (CL), which is required for mitochondrial bioenergetics and the induction of apoptosis. The precursor phosphatidic acid (PA) for CL synthesis is produced in the ER and transported into the mitochondria via the MAM platform.^63^

Mitochondrial‐associated membrane is also enriched with multiple lipid transport proteins and biosynthetic enzymes. For example, fatty acid‐CoA ligase 4 (FACL4) mediates the linkage of fatty acids to cholesterol metabolites and coenzyme A,^64^ ACAT1/SOAT1 is involved in cholesterol metabolism and transport,^65^ and diacylglycerol *O*‐acyltransferase 2 (DGAT2) participates in the regulation of cholesterol esterification and triglyceride synthesis.^66^ Therefore, it is important to explore the mechanisms by which proteins regulate lipid transport while participating in biosynthesis. As mentioned above, ERMES is a complex of five subunits, of which Mmm1, Mdm12 and Mdm3 share a shape memory polymer (SMP) structural domain, characterized by the presence of a hydrophilic pocket in the phospholipid‐lipid binding region.^67^ The Mmm1–Mdm12 complex generates a continuous 210‐Å‐long hydrophobic tunnel that facilitates phospholipid transport.^68^ Taken together, lipid metabolism is a key function of MAM and is important for overall cellular health.

### 4.2 Roles of lipid transport between ER‐mitochondria in cancer

Cancer cells often exhibit altered cholesterol levels, which may favour resistance to apoptosis and metabolic reprogramming.^69^, ^70^ The lipid composition of IMM has been reported to be altered in cancer cells, with some solid tumours displaying increased levels of cholesterol and phospholipids with fewer unsaturated acyl chains.^71^ In this section, we provide an overview of lipid‐related proteins in MAM that have been implicated in cancer development.

Evidence suggests that mitofusin 2 (MFN2) is a mitochondrial membrane protein that links the ER member with the mitochondria^72^ and plays a relevant role in maintaining mitochondrial metabolism and energy homeostasis.^73^ Recently, MFN2 was found to specifically bind PS and extract it from the MAM structural domain, facilitating subsequent PE synthesis.^74^ Various cancer cells show low MFN2 expression, and MFN2 expression is negatively associated with tumour prognosis in patients with breast cancer and HCC.^75^, ^76^ It has been found that MFN2 deficiency reduces PS metastasis and alters phospholipid metabolism, resulting in ER stress and promoting hepatocarcinogenesis in a non‐alcoholic steatohepatitis (NASH) mouse MFN2 re‐expression experiment.^74^ In addition, the overexpression of MFN2 in CRC cells has been found to decrease cell proliferation and induce spontaneous apoptosis.^77^

Interestingly, the above‐mentioned alterations in MAM‐resident protein activity may affect the concentration of intracellular signalling molecules involved in cancer development. ACAT1 is a resident enzyme in the ER that converts cholesterol to cholesteryl esters for storage and links oncogenic signals to energy metabolism.^78^ Fan et al.^79^ demonstrated that ACAT1 can act as an upstream acetyltransferase of the pyruvate dehydrogenase complex (PDHA and PDP1), both of which are important for promoting glycolysis in NSCLC cells (H1299 cell line) and subsequent tumour growth. Moreover, ACAT1‐mediated cholesterol ester (CE) accumulation is positively correlated with the proliferation and metastasis of cancer cells and a poor prognosis in prostate cancer.^80^ Specifically, reduced ACAT1‐catalysed CE synthesis leads to free cholesterol accumulation, decreased LDL receptor levels and reduced essential fatty acid uptake, thereby impairing cell proliferation and tumour growth *in vivo*. On the other hand, CE has been shown to be a major component of lipid droplets (LDs) in cancer cells and the formation of LD is associated with cancer proliferation and progression.^81^ Geng et al.^82^ showed that cholesterol esterification and LD formation are characteristic of glioblastoma, and elevated LD levels in patients with glioma are associated with cancer progression and poor survival.

Steroidogenesis Acute Regulatory (STAR) and ATPase family AAA‐domain containing protein 3A (ATAD3a) (localized to MAM) are two essential proteins that regulate cholesterol entry into mitochondria.^83^ The overexpression of STARd4 is associated with increased mitochondrial cholesterol content, whereas STAR knockdown enhances the sensitivity of HCC cells to chemotherapeutic agents.^84^ High levels of ATAD3a expression may facilitate aggressiveness in cancer cells by increasing membrane cholesterol and is related to a poor prognosis in patients with breast cancer.^85^ In addition, another study revealed that ATAD3A knockdown in mouse testicular stromal tumour cells (MA‐10) resulted in a significant reduction in steroidogenesis.^86^ Thus, ATAD3A is considered to be an oncoprotein associated with cholesterol metabolism.

Another MAM‐resident protein, oxysterol‐binding protein‐related protein 5 and 8 (ORP5/8), mediates the non‐vesicular transport of PS lipids from the ER to the mitochondria.^87^ Several studies have linked increased OPR expression to cancer progression. In particular, ORP5 expression is associated with an increased invasion and metastasis of cancer cells, possibly because of its metastatic function in ER‐mitochondria contact sites (EMCS).^88^, ^89^ For example, ORP5 promotes the proliferation and motility of cervical cancer cells (HeLa cells), which is dependent on its functional oxygen sterol‐binding protein‐related domain (ORD). The deletion or replacement of key residues located within ORP5‐ORD that interact with lipids can disrupt cell proliferation, migration and invasion.^90^ Collectively, these findings demonstrate that the dysregulated expression of MAM‐related proteins has a direct impact on lipid metabolism, which, in turn, affects cancer progression. Thus, the lipid composition of ERMES may affect lipid homeostasis and ER/mitochondrial function, which are critical for regulating apoptotic signalling in tumours.

## 5 AUTOPHAGY AND MAM

### 5.1 Autophagosomes form at the ERMES platform

Autophagy is a lysosome‐mediated intracellular material recycling system that plays an essential role in maintaining cellular homeostasis and integrity and is an alternative approach of providing energy in the presence of nutritional deficiencies.^91^, ^92^ Starvation induces mitochondrial elongation, which allows mitochondria to efficiently produce energy and avoid autophagic degradation. Notably, the interaction between ER and mitochondria is involved in this process.

Three‐dimensional electron tomography studies revealed that vital proteins involved in autophagosome formation, such as autophagy‐related 14 (ATG14) and autophagy‐related 5 (ATG5), are recruited to the EMCS after starvation.^93^ In brief, during the initiation of phagocytic expansion, starvation triggers the translocation of ULK1 to MAMs and then activates the phosphorylation of the downstream autophagy effector protein Beclin‐1. This process occurs in the PI3P‐rich substructural domain of ER. Next, PI3P promotes a variety of proteins, such as ATG14, which gradually approach and localize to MAM under starvation conditions.^94^ In parallel, ATG5 (a marker of autophagosome formation) is transferred to MAM until its formation is complete.^95^ During phagocytic expansion, ATG2A is translocated from MAMs to phagocytes to enhance autophagic flux, depending on the OMM components of the mitochondria: TOM40 and TOM70.^96^ Moreover, the depletion of a major regulator of MAM, MFN2, impairs membrane formation or blocks fusion during autophagy.^97^

In addition, several MAM‐resident and tethered proteins have been reported to regulate autophagy. For example, the regulation of tethering via the overexpression or downregulation of VAPB‐PTPI51 can impair or stimulate autophagy, respectively. In turn, these effects regulate autophagy by mediating Ca^2+^ transfer from the ER to mitochondria.^98^ Altogether, autophagy has been found to be associated with MAM, and these dynamic regulations further reveal that MAM plays a critical role in determining the cell fate.

### 5.2 Roles of autophagy‐mediated by MAM in cancer

Accumulating evidence has revealed that autophagy plays a dual role in tumorigenesis, that is, either promoting or inhibiting tumour cell survival.^99^ During tumour development, enhanced autophagy enters the ATP synthesis or biosynthesis pathways by recirculating intracellular components, thereby maintaining cellular energy homeostasis and regulating the secretion of oncogenic factors. In contrast, autophagy‐associated tumour suppressors can exert anticancer effects by maintaining cellular integrity and mitigating important cellular functions of cell damage under metabolic stress conditions, such as scavenging ROS and damaged mitochondria.^100^ In this section, we have summarized the role of MAM‐regulated autophagy in carcinogenesis.

As mentioned previously, some oncogenes and tumour suppressors found in MAM are key regulators of autophagy.^101^ In particular, the induction of autophagy in tumour cells is controlled by MAM‐associated promyelocytic leukaemia (PML) and p53. The tumour suppressor PML forms a multiprotein complex with Akt, IP3R3 and protein phosphatase 2 A (PP2A) on MAM and controls autophagy between the ER and mitochondria in a Ca^2+^ transfer‐dependent manner.^102^ Missiroli et al.^103^ found that PML from MAMs acts on Ca^2+^ transfer from the ER to the mitochondria, thereby regulating apoptosis and autophagy in cancer cells. Thus, the stimulation of autophagy directly leads to reduced mitochondrial respiration, and the absence of PML confers resistance to apoptotic stimuli and metabolic stress, thereby promoting cell survival in the tumour environment. In addition, p53 maintains the proper localization of PML in the MAM structural domain, whereas PML may function as a major autophagy downstream of p53, which underlies the inhibition of autophagy.^104^

In addition, blocking ER‐mitochondria Ca^2+^ transport in tumour cells induced the local autophagy activation of AMPK in MAM, which subsequently increased the expression level of Beclin 1 (BECN1).^105^ Chhipa et al.^106^ explored the relationship between AMPK and BECN1 in prostate cancer cells and found that, in the presence of androgen deficiency and hypoxia, an enhanced AMPK response was necessary to trigger autophagy, which provides a survival advantage for human prostate cancer cells (LNCaP cell line). In addition, BECN1 is an important downstream target of AMPK for initiating autophagy cascades in prostate cancer cells. Therefore, if chemical inhibitors of AMPK or BECN1 are utilized to block autophagy, a greater number of cancer cells will die from apoptosis.

Moreover, BECN1 is a key autophagy regulator gene whose expression deletions are observed in multiple tumours, such as ovarian and prostate cancers and is thus considered a haploinsufficient tumour suppressor gene.^107^, ^108^ BECN1 can either promote or inhibit autophagy by directly interacting with AMBRA1 or BCL‐2.^109^ BCL‐2, an anti‐autophagy protein, interacts with BECN1 to inhibit autophagy. The expression of BECN1 in breast cancer tissues has been found to be negatively correlated with that of BCL‐2. Thus, BECN1 exerts an inhibitory effect on breast cancer progression by interacting with BCL‐2.^110^ In contrast to BCL‐2, AMBRA1 stimulates autophagy. BCL‐2, located in the mitochondria, can directly bind AMBRA1, thus preventing AMBRA1 from binding to BECN1. After autophagy induction, this interaction is dissociated and AMBRA1 can replace BCL‐2 to activate BECN1.^109^ In CRC, AMBRA1 and BECN1 interact with rapamycin‐treated SW620 cells, indicating that AMBRA1 regulates autophagy in CRC cells by interacting with BECN1.^111^ Taken together, these observations reveal that autophagy prevents malignant transformation by maintaining cellular and biological homeostasis in the presence of tumorigenic risk conditions and that the interaction between oncogenic kinases and autophagy proteins is an important mechanism involved in tumorigenesis.

## 6 ENDOPLASMIC RETICULUM STRESS AND MAM

### 6.1 Regulation of ER stress signalling occurs in the MAM platform

The correct folding for the *de novo* synthesis of proteins is one of the most energy‐demanding processes in cells. However, when ER stress triggers the accumulation of unfolded proteins that cannot be catabolized, the UPR (a conserved intracellular pathway) turns on and initiates pro‐apoptotic signalling, ultimately leading to apoptotic cell death.^112^ In general, UPR is primarily a pro‐survival response to restore ER homeostasis.^113^ There is a strong connection between the MAM components and UPR.^114^ Undoubtedly, MAM has been confirmed to be a hotspot for the transfer of stress signals from the ER to mitochondria via the UPR, particularly in the context of the loss of ER protease homeostasis.^115^

Three key ER stress sensors modulate the signalling events associated with the loss of ER homeostasis by directly affecting the expression of proteins localized to the MAM, including protein kinase‐like ER kinase (PERK), IRE1 and ATF6.^116^ Under normal conditions, they remain in an inactive state. When ER stress occurs, they are activated by dissociation from GRP78/BIP, the major regulatory molecular chaperone of the UPR, thus contributing to the correct folding of the nascent polypeptide.^117^ ER stress can affect redox‐signalling by regulating ER‐mitochondrial Ca^2+^ channels and MAM members.^28^ Through functional and subcellular isolation, Verfaillie et al.^118^ found that PERK is an important member of the MAM family and plays a tethering role in ERMES. Another key protein, MFN2, interacts directly with PERK, and the PERK‐MFN2 pair is required for the ER stress‐mediated pathway.^119^ PERK also induces Eif2 phosphorylation and the subsequent activation of ATF4, which, in turn, induces an increase in the expression of protein‐folding factor genes, such as CHOP and GRP75, thereby regulating ER homeostasis.^94^, ^120^ After ER stress induction, ATF6 response genes are rapidly activated, such as the molecular chaperones (Bip and GRP94) and XBP1 mRNA. Evidence indicates that XBP1 can be converted into a stable transcription factor through the nucleic acid endonuclease activity of IRE1, which is involved in the regulation of protein folding.^121^ For the IRE1 pathway, it has been found to be the last activated branch of the UPR. In response to ER stress, IRE1 promotes dimerization by interacting with the MAM‐resident ER chaperone Sig‐1R, which functions as a Ca^2+^ receptor and segregates from GRP78 upon ER Ca^2+^ depletion to facilitate IP3R‐mediated Ca^2+^ transfer to the mitochondria.^95^ Moreover, if ER stress persists, IRE1 can activate XBP1s to initiate pro‐apoptotic protein synthesis, thereby promoting apoptosis.^122^

### 6.2 Roles of ER stress at MAM in cancer

In cancer, ER signalling pathways are frequently dysregulated, thereby promoting cancer cell metabolism. The UPR may play a key role in the proliferation, metastasis and survival of cancer cells. Importantly, MAM is a critical hub for cancer prosperity and cell death and provides a platform for the regulation of signalling events associated with the loss of ER homeostasis.

It has been reported that PERK protein is detected in several cancer cells, which can limit the progression of the cell cycle through oxidative DNA damage checkpoints. JI017 (an herb mixture composed of *Aconitum carmichaelii*, *Angelica gigas* and *Zingiber officinale*) exerts anticancer effect in prostate cancer cells via ROS‐mediated ER stress.^123^ Briefly, JI017 generates ROS and induces ER stress. Stress conditions induce the dissociation of GRP78/BIP (sensor key regulatory protein) from the IRE1α and PERK sensors, and subsequently activate sensor proteins, leading to eIF2α activation. Meanwhile, IRE1α phosphorylates JNK1. JNK signalling and UPR sensors activate CHOP. Sustained ER stress can trigger apoptosis. Notably, this apoptotic stress is mediated through the mitochondrial pathway, specifically involving BCL‐2 family members, pro‐apoptotic Bax and BH3 proteins. During mitochondrial degradation, changes in apoptotic factors lead to the loss of mitochondrial membrane potential (ΔΨm), and small molecules, such as cytochrome C, are subsequently released through the mitochondrial pore and the activation of the mitochondrial apoptotic pathway through cleaved caspase‐3 and ‐9 to induce apoptosis. ATF3, a downstream factor of PERK, is also activated under ER stress conditions, which further downregulates the expression of the pro‐tumour chemokine EGR‐1 and affects colon cancer cell survival.^124^ On the other hand, in glioblastoma multiforme, PERK is responsible for ceramide production, which is essential for Ca^2+^ induction and ROS generation, ultimately stimulating cellular autophagy and death.^125^

Most cancers are continuously exposed to a variety of stresses, during which PERK signalling and the region downstream of the integrated stress response (ISR) are activated, enabling cancer cells to survive hypoxia and nutrient starvation.^126^ Conversely, the ablation of the PERK signalling pathway or ISR causes ROS production, which inhibits tumour growth via oxidative DNA damage.^127^ In ISR, different protein kinases phosphorylate Eif2α, which inhibits overall protein translation and synthesis, thereby reducing the amount of protein that enters the ER.^128^ Simultaneously, an increase in ATF expression has been reported. The PERK‐ATF4 axis induces VEGF (a proangiogenic mediator) expression, which stimulates endothelial cell survival and angiogenesis in the unfavourable microenvironment. Experimental evidence has shown that the knockdown of PERK and ATF4 in breast cancer (MCF7) and HNSCC cell lines (UM‐SCC‐81B) can slow tumour growth and reduce tumour vascular density.^129^ In addition, the suppression of ATF4 expression in the context of amino acid deprivation and glucose deficiency inhibits tumour survival and proliferation in the HeLa human cervical cancer cell line.^130^ Taken together, cancer cells are able to adapt to hypoxia, oxidative stress and nutritional starvation via improved ISR‐ and PERK‐mediated redox and metabolic homeostasis.

Furthermore, under acute ER stress conditions, Sig‐1R (a chaperone molecule of MAM) counteracts the ER stress response by translocating to the ER, thereby reducing apoptosis.^131^ Higher levels of Sig‐1R expression were reported in breast cancer cells with metastatic potential.^132^ In addition, Sig‐1R antagonists induce UPR activation and ER stress in adenocarcinoma cell lines, such as breast adenocarcinoma (MCF7) and prostate adenocarcinoma (PC3), which leads to the induction of autophagy and subsequent apoptotic activation, suggesting that UPR and autophagy induction mediate the cell protective effect of Sig‐1R in cancer cells.^133^ As mentioned above, another MAM chaperone protein, GRP78, can bind to the ER antiapoptotic factor CLU during ER stress to promote its redistribution in mitochondria, thereby reducing the deleterious effects caused by ER stress and contributing to the induction of prostate cancer cell survival.^134^ Thus, MAM has broad implications as a control site for UPR and mitochondrial function and is expected to participate in cancer progression by affecting tumour homeostasis and bioenergetics.

## 7 MITOCHONDRIAL‐ASSOCIATED MEMBRANE AND CANCER THERAPY

**FIGURE 3:**
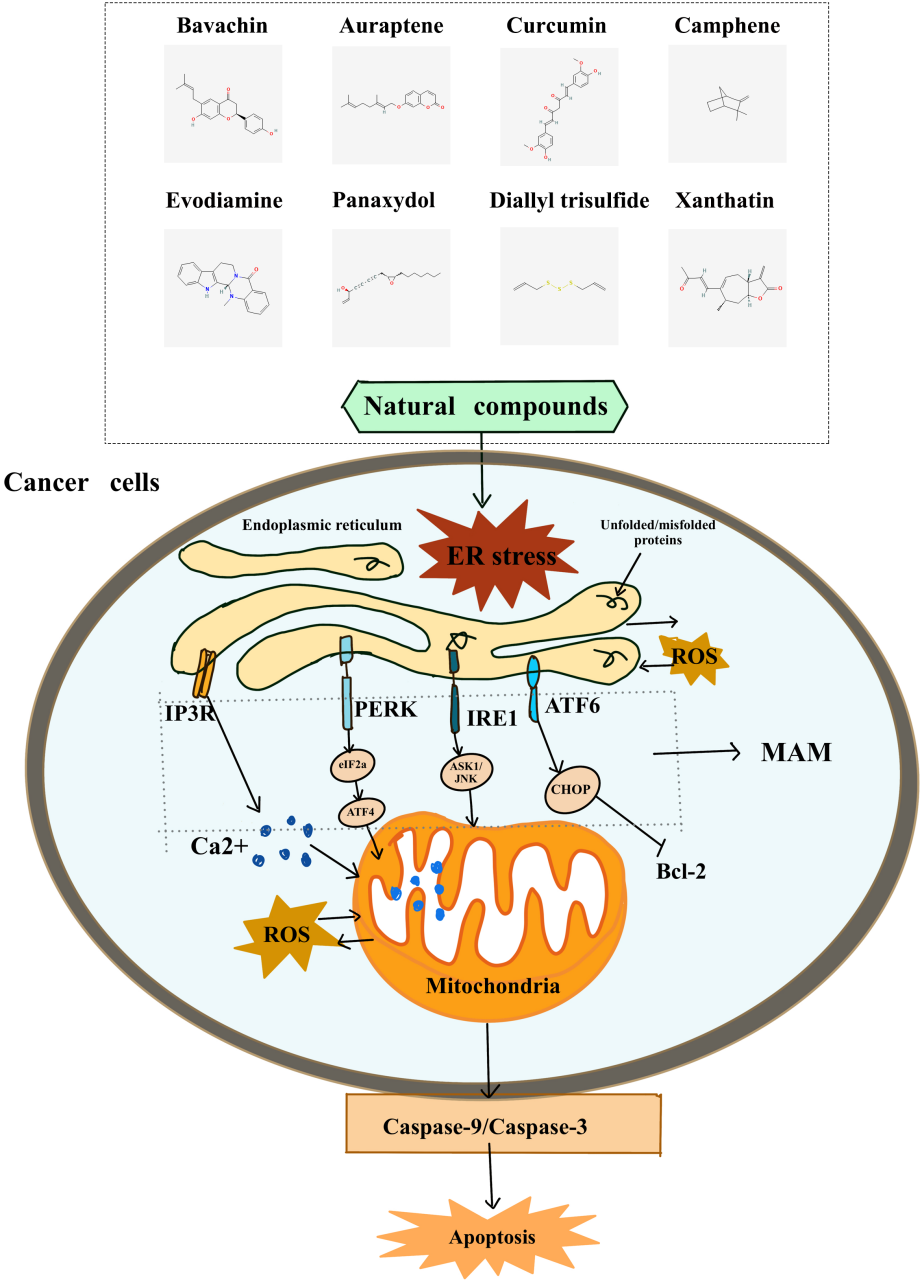
Natural compounds or MAM‐related proteins induce apoptosis associated with endoplasmic reticulum stress via the mitochondrial pathway to affect the nature of cancer.

In the above section, we summarized the key roles of MAM in cancer progression from multiple perspectives, including Ca^2+^ signalling transfer, lipid synthesis, cellular autophagy and ER stress. In the current section, we focus on natural compounds or MAM‐related proteins and explore how they induce apoptosis associated with ER stress through mitochondrial pathways, which, in turn, affects the nature of cancer. ER stress‐related apoptosis in MAM, triggered by natural compounds, is shown in Figure 3.

### 7.1 Bavachin

Bavachin is a flavonoid extracted from the fruit of *Bacopa monniera* with various functions, such as anti‐inflammatory, hypolipidemic and hypocholesterolemic effects.^135^ A previous study revealed that bavachin has anticancer properties; for example, it can inhibit human liver cancer cells by increasing ROS accumulation and inducing apoptosis.^136^ Furthermore, bavachin can also affect the interaction between the ER and mitochondria and can serve as a candidate drug for the treatment of human placental choriocarcinoma.^137^ Specifically, bavachin inhibits choriocarcinoma cell proliferation by modulating the electron transport chain complexes and OXPHOS to alter the metabolic phenotype, as well as by causing Ca^2+^ transport disruption and ER stress accompanied by mitochondrial membrane potential (MMP) depolarization.

### 7.2 Auraptene

Auraptene, originally isolated from citrus fruits, has been reported to exhibit anti‐inflammatory, antioxidant and immunomodulatory effects in animal models.^138^, ^139^ Interestingly, auraptene has also been shown to exert chemopreventive activity against chemo‐induced carcinogenesis in rodents.^140^ Jun et al.^141^ showed that auraptene extracted from *Zanthoxylum schinifolium* displayed strong apoptotic activity in the Jurkat T‐cell line (human acute leukaemia). Briefly, apoptosis induced by auraptene is initiated by ER stress, triggered by the activation of caspase‐8, caspase‐12 and JNK, followed by the activation of caspase‐3 and ‐9 to induce pro‐Jurkat T‐cell apoptosis. They also indicated that this apoptotic effect could be inhibited by Bcl‐xL.

### 7.3 Curcumin

Curcumin is the main bioactive compound of *Curcuma longa L*. and has been confirmed to prevent and treat multiple tumours, including pancreatic cancer,^142^ oral cancer^143^ and colorectal cancer.^144^ The anticancer effect of this compound may be attributed in part to its structural properties; that is, the presence of o‐methoxyphenol and methylene hydrogen are responsible for providing an electron/hydrogen to reactive oxygen species, thereby stimulating antioxidant activity.^145^ de Oyanguren et al.^146^ used image flow cytometry to study the cellular localization and anticancer mechanism of curcumin in detail and found that curcumin is closely related to ER stress and mitochondrial homeostasis. Curcumin primarily acts on the ER and is associated with the UPR response, ER swelling and calcium release. Increased GPR78/Bip expression has been observed in curcumin‐treated HCC cells and is required for stress‐induced autophagy. In contrast, curcumin treatment increased Bax/Bcl‐2 in the HCC cell line (Huh‐7) and led to leakage of mitochondrial membranes, which, in turn, caused mitochondrial instability and apoptosis. Moreover, curcumin was reported to inhibit SERCA expression in HCC cells, which induced an increase in the cytosolic levels of calcium.

### 7.4 Camphene

Camphene is a bicyclic monoterpene found in many essential oils, including cypress oil, citronella oil and bergamot oil.^147^ Camphene extracted from the crude volatile oil of *Piper cernuum Vell*. has been reported to exert anti‐tumour activity, and markers of apoptosis were detected in camphene‐induced melanoma cells.^148^ Camphene functions induce cellular ER stress and Ca^2+^ release through multiple endogenous pathways, resulting in decreased mitochondrial membrane punctuation and the upregulation of caspase‐3 activity.

### 7.5 Evodiamine

Evodiamine has been isolated from various medicinal plants, such as the herbal plant *Evodiae fructus* and is an active ingredient in many traditional pharmaceutical preparations.^149^ Previous studies have reported that evodiamine has biological effects, such as anti‐tumour and anti‐injury. For example, evodiamine inhibits cell invasion and lung metastasis in colon cancer.^150^ Chen et al.^151^ described the mechanism of evodiamine‐induced apoptosis in human ovarian cancer cells from the perspective of ER stress. In their study, they revealed that evodiamine disrupts mitochondrial membrane potential by activating PERK (ER stress sensor) and JNK, which results in the apoptosis of ovarian cancer cells.

### 7.6 Panaxydol

Panaxydol is a polyacetylene compound extracted from *Panax ginseng* roots that suppresses the growth of cancer cells.^152^, ^153^ Mechanistic studies have revealed that EGFR activation and ER stress regulate panaxydol‐induced apoptosis and that panaxydol suppresses prostate tumour growth in a mouse tumour model.^154^ PERK, but not ATF6 or IRE1a, plays a role in panaxydol‐induced apoptosis. Indeed, during the induction of apoptosis in cancer cells, panaxydol was found to trigger the pro‐apoptotic transcription factor CHOP through the PERK/eIF2a/ATF4 signalling pathway, which subsequently activated Bim (a pro‐apoptotic transcriptional target of CHOP), thereby enabling apoptotic signalling from the ER to the mitochondria.

### 7.7 Diallyl trisulfide

Diallyl trisulfide (DATS) is an organosulfur compound in garlic that exerts therapeutic effects in a variety of cancers.^155^ DATS‐mediated apoptosis in basal cell carcinoma (BCC) is associated with intracellular ROS accumulation and the disruption of the mitochondrial membrane potential.^156^ The mechanism of apoptosis can be explained from three aspects: first, DATS increases ROS levels and causes DNA damage; second, DATS induces ER stress‐related molecules (such as Bip/GRP78) and Ca^2+^ mobilization, leading to caspase‐4 and ‐9 activation; and finally, DATS induces an increase in cellular pro‐apoptotic factors (Bax) and a decrease in antiapoptotic factors (Bcl‐2 and Bcl‐xl), which, in turn, leads to the loss and release of mitochondrial proteins, subsequently triggering caspase‐dependent apoptosis. Taken together, DATS exerts its chemopreventive effects in BCC cells via ER stress and mitochondrial pathways, which are considered to be potential anticancer agents in skin cancer.

### 7.8 Grape seed proanthocyanidins

Grape seed proanthocyanidins (GSPs), a mixture of polyphenols extracted from grape seeds, exhibit anticancer activity.^157^ Chen et al.^158^ indicated that treatment with GSPs inhibited cervical cancer cell activity and slowed tumour growth in a mouse model of cancer while increasing the number of apoptotic cells *in vivo*. This pro‐apoptotic mechanism is triggered through the mitochondrial pathway, which is specifically manifested by the downregulation of Bcl‐2 (antiapoptotic protein) and upregulation of Bak‐1 (pro‐apoptotic protein), resulting in the loss of mitochondrial membrane potential and increased caspase‐3 activity.

### 7.9 Xanthatin

Xanthatin is a natural sesquiterpenoid lactone purified from flavberine that shows prominent anti‐tumour activity against multiple cancers.^159^ In glioma cells and xenografted nude mice, xanthatin treatment has been reported to induce apoptosis in cancer cells.^160^ Specifically, xanthatin treatment activates the expression levels of ER stress‐related markers, such as CHOP and then regulates caspase‐3 expression, suggesting that xanthatin inhibits tumour growth by inducing ER stress‐associated UPR pathways. This evidence highlights the role of natural compounds in ER‐mitochondrial communication. These compounds improve multiple ER stress‐mediated signalling pathways, and in cancer, they can induce apoptosis via the ER stress‐mediated mitochondrial apoptosis pathway.

## 8 CONCLUSION

In conclusion, MAM represents a platform for multiple intracellular signalling pathways and plays crucial roles in the regulation of Ca^2+^ transfer, lipid metabolism, autophagy and ER stress. Interestingly, these signalling pathways are closely associated with both pro‐ and anticancer processes. Furthermore, Ca^2+^ exchange and apoptotic signalling involved in MAM are altered during carcinogenesis, which promotes cancer cell proliferation, survival and metabolic reorganization. Increasing evidence highlights the important role of MAM in cancer development; thus, targeting MAM‐specific resident proteins is expected to be a new strategy for cancer therapy. However, different ER‐mitochondrial contact sites may also exist in different cell types. In addition, focusing on these MAM functions in cancer cells without affecting normal cells represents be a major challenge. Thus, we believe that further exploration of the signalling pathways involved in MAM will provide new insights into cancer treatment.

## AUTHOR CONTRIBUTIONS

**Xi Yang:** Validation (equal); visualization (equal); writing – original draft (equal); writing – review and editing (equal). **Jing Zhuang:** Validation (equal); visualization (equal); writing – original draft (equal); writing – review and editing (equal). **Weilong Song:** Data curation (equal); formal analysis (equal); investigation (equal); visualization (equal). **Wangjie Shen:** Data curation (equal); formal analysis (equal); investigation (equal); validation (equal). **Wei Wu:** Data curation (equal); formal analysis (equal); investigation (equal); validation (equal). **Hong Shen:** Conceptualization (equal); methodology (equal); project administration (equal); supervision (equal); validation (equal); writing – review and editing (equal). **Shuwen Han:** Conceptualization (equal); methodology (equal); project administration (equal); supervision (equal); validation (equal); writing – review and editing (equal).

## FUNDING INFORMATION

This work was supported by Public Welfare Technology Application Research Program of Huzhou (numbers 2021YZ22 and 2021YZ30), Zhejiang Provincial Natural Science Foundation (number Q23H160033) and Zhejiang Medical and Health Technology Project (number 2022KY1220).

## CONFLICT OF INTEREST STATEMENT

The authors declare that no potential conflicts of interest exist.

## Data Availability

Not applicable.
